# Perception of integrating an AI teaching module into medical education curriculum

**DOI:** 10.3389/fmed.2026.1774935

**Published:** 2026-03-03

**Authors:** Jude Jamjoom, Faisal Alkhwaiter, Ghedaa Armosh, Nour Alfarra, Shahad Murshid, Aya Tamim, Hani Tamim, Noara AlHusseini, Selwa Al-Hazzaa

**Affiliations:** 1 College of Medicine, Alfaisal University, Riyadh, Saudi Arabia; 2 College of Biological Sciences, University of Guelph, Guelph, ON, Canada; 3 Department of Internal Medicine, Clinical Research Institute, American University of Beirut Medical Center, Beirut, Lebanon; 4 King Abdulaziz City for Science & Technology (KACST), Riyadh, Saudi Arabia

**Keywords:** artificial intelligence (AI), medical curricula, medical curriculum, medical education, educational outcomes

## Abstract

**Background and aims:**

Artificial intelligence (AI) is evolving into a revolutionary tool as medical education rapidly adapts to meet the demands of modern healthcare. This study examined the perceptions of faculty members, teaching assistants, and medical students regarding the integration of AI teaching modules into the undergraduate medical curriculum at Alfaisal University in Riyadh, Saudi Arabia.

**Methods:**

A cross-sectional questionnaire-based survey was conducted among 201 participants (68 faculty members, 16 teaching assistants, and 117 medical students). The survey collected demographic data (age, gender, nationality, academic role, and faculty rank or student year of study) and explored perceived advantages (e.g., innovation, efficiency, accuracy), disadvantages (e.g., workload, resistance, job replacement, overreliance on technology), and views on the appropriate stage for introducing AI in the curriculum. Responses were measured on a five-point Likert scale and analyzed using descriptive and inferential statistics.

**Results:**

The majority of respondents expressed favorable perceptions of AI integration, highlighting its potential to inspire innovation, improve efficiency, enhance clinical precision, and broaden medical specialties. Over half (55.7%) recommended introducing AI during preclinical years, while 32.8% preferred the clinical years.

**Conclusion:**

The findings demonstrate strong support for the early integration of AI into Alfaisal University’s medical curriculum. These insights provide evidence to guide curriculum development and prepare future medical professionals for AI-driven practice.

## Introduction

Medical education has been rapidly evolving, with various innovative methods and tools being employed to enhance knowledge delivery and the quality of teaching. Techniques such as team-based learning, simulation-based training, and other revolutionary approaches have significantly impacted medical training (1, 2). Among these advancements, the integration of artificial intelligence (AI) into medical education has seen exponential growth. AI has demonstrated its potential to facilitate more efficient research processes, enable personalized learning experiences, improve diagnostic accuracy through simulation, and offer sophisticated assessment tools (3).

Recent studies have shown that interest in using AI in medical education is growing. AI is generally viewed favorably by educators and students, who recognize that it can improve various aspects of medical education. These developments are considered crucial for training future physicians to operate in healthcare settings that are becoming increasingly reliant on technology (4, 5). Furthermore, a study from Kuwait University shows that many students identified the coming importance of AI in healthcare, underscoring the urgency of incorporating AI into medical curricula. This opinion is significant because it indicates a wave of support for AI-based educational reforms from the future workforce (6).

Despite the optimism surrounding the use of AI in medical education, concerns persist. A study conducted among physicians in Jeddah, Saudi Arabia, highlighted mixed feelings about AI. While many acknowledged its accuracy and efficiency, they also expressed concerns about job security and the potential for an emotional disconnect in patient care (7). A key challenge is that many medical students and instructors currently learn about AI through informal sources, such as the media, rather than through structured, formal education (8, 9). Additionally, a study conducted in Palestine found that medical students lacked sufficient training in AI. This disparity highlights the potential disconnect between the demands of the healthcare professions and the current approach to education (10).

The readiness of medical professionals for AI varies significantly. A study of AI readiness among Saudi medical and dental professionals found generally poor levels of preparedness, highlighting the urgent need for comprehensive educational programs that incorporate AI and its applications. This gap in preparation highlights the need to implement specialized educational activities catering to unique needs and settings (11).

Although there is extensive literature on incorporating AI into medical education, there is limited agreement on what and how to teach and incorporate AI in medical education (12). This study aimed to explore perceptions of integrating AI-powered medical software into the medical education curriculum at a private university in Saudi Arabia. By examining these perceptions, the research seeks to provide insights that can guide the effective integration of AI technologies into medical education, ensuring that both faculty and students are well-prepared to utilize these advancements in their future professional practices.

## Methods

The study was conducted at the College of Medicine, Alfaisal University in Riyadh, Saudi Arabia, using a cross-sectional, questionnaire-based design. Alfaisal University is a private, non-profit institution founded in 2002, with the College of Medicine being one of its founding colleges. The approximate population size is 100, 60, and 1,500 for faculty, teaching assistants, and students, respectively. Ethical approval was obtained from the Alfaisal Institutional Review Board (IRB approval number: IRB20332). Participation was voluntary, and informed consent was obtained electronically prior to survey completion.

Eligible participants included medical students across all years of study, teaching assistants, and faculty members engaged in teaching, curriculum development, or academic activities at the College of Medicine. Individuals who did not meet these criteria or declined to provide consent were excluded. A total of 201 participants completed the survey, consisting of 68 faculty members, 117 students, and 16 teaching assistants. Therefore, the response rate is 68/100 is 68% for faculty, 16/60 is 27% for teaching assistants, and 117/1500 is 8% for students.

As for sampling, Participants were recruited using a voluntary response sampling method through an email invitation sent to all eligible subjects (faculty, teaching assistants, and students). All responses from the invitation were included in the analysis.

A structured, self-administered questionnaire was developed using Google Forms, adapted from previously validated surveys to ensure content validity and comparability with international literature (7, 9, 13–19). The questionnaire included four domains. The first collected demographic information such as age, gender, nationality, role at Alfaisal University, academic rank or year of study, specialty, and years of experience. The second domain examined perceived advantages of integrating AI into medical education, including innovation, efficiency, and clinical accuracy. The third domain assessed disadvantages and challenges, including increased workload, resistance to new technology, resource limitations, fear of job replacement, and overreliance on technology. The fourth domain addressed perceptions of the future role of AI in medical education and asked participants to indicate the most appropriate stage for integration (preclinical years, clinical years, residency, or fellowship). All items in these domains were rated on a five-point Likert scale ranging from 1 (*strongly disagree*) to 5 (*strongly agree*). Negatively worded items (Q16–Q22) were reverse-coded so that higher scores indicated more positive perceptions.

The survey was distributed electronically via institutional email between September and October 2024. Responses were anonymized prior to analysis. Data were exported into SPSS version 29 (IBM Corp., Armonk, NY, USA) for analysis. Descriptive statistics were used to summarize demographic variables. Continuous variables were reported as means and standard deviations, while categorical variables were presented as frequencies and percentages. Comparisons across roles (faculty, students, and teaching assistants) were performed using Chi-square tests for categorical variables and one-way ANOVA for continuous variables. An overall perception score was calculated by averaging Likert-scale responses and rescaling the mean to a 0–100 scale. Higher values indicated better perception. Multiple linear regression was performed to identify predictors of the overall perception score. Results are reported as *β* coefficients with 95% confidence intervals (CIs). multiple logistic regression was used to assess predictors of positive perception, defined as a score ≥60. Results are reported as odds ratios (ORs) with 95% CIs. Nationality was collapsed into two categories (Saudi vs. Non-Saudi) to ensure adequate sample sizes for analysis. Internal consistency of the scale was assessed by calculating the Cronbach’s alpha. Statistical significance was set at *p* < 0.05. In addition, R version 2025.05.1 + 513 (R Foundation for Statistical Computing, Vienna, Austria) was used to generate figures for visualization of Likert responses and stage preferences.

## Results

A total of 201 participants completed the survey, comprising 68 faculty members (33.8%), 117 students (58.2%), and 16 teaching assistants (8.0%). Table 1 summarizes the baseline characteristics. More than half of the respondents (56.7%) were between 18 and 24 years of age, 16.4% were between 25 and 44 years old, and 26.9% were 45 years or older, with statistically significant differences across the groups (*p* < 0.001). Gender distribution was nearly equal (48.3% male and 51.7% female), although males were more prevalent among faculty (72.1%) compared to students (36.8%) (*p* < 0.001). Nearly one-third of participants were Saudi nationals (29.4%), while the majority (70.6%) were non-Saudi (*p* < 0.001). Among students, all years of study were represented, with the largest proportion being third-year students (23.9%). Within the faculty group, professors and assistant professors each accounted for 35.3%, while associate professors and lecturers/senior lecturers represented 17.6 and 11.8%, respectively. Most faculty (79.4%) reported more than 10 years of professional experience.

**Table 1:** Baseline characteristics of study participants by position.

Variable	All *N* = 201	Faculty *N* = 68	Student *N* = 117	Teaching assistant *N* = 16	*p*-value
Demographics					
Age					
18–24	114 (56.7%)	2 (2.9%)	109 (93.2%)	3 (18.8%)	<0.001
25–44	33 (16.4%)	12 (17.6%)	8 (6.8%)	13 (81.3%)	
45+	54 (26.9%)	54 (79.4%)	0 (0.0%)	0 (0.0%)	
Gender					
Male	97 (48.3%)	49 (72.1%)	43 (36.8%)	5 (31.3%)	<0.001
Female	104 (51.7%)	19 (27.9%)	74 (63.2%)	11 (68.8%)	
Nationality					
Saudi	59 (29.4%)	38 (55.9%)	21 (17.9%)	0 (0.0%)	<0.001
Non-Saudi	142 (70.6%)	30 (44.1%)	96 (82.1%)	16 (100.0%)	
Student–year					
Intern	5 (2.5%)	–	5 (4.3%)	–	N/A
Year 1	22 (10.9%)	-	22 (18.8%)	–	
Year 2	15 (7.5%)	-	15 (12.8%)	–	
Year 3	48 (23.9%)	–	48 (41.0%)	–	
Year 4	19 (9.5%)	–	19 (16.2%)	–	
Year 5	8 (4.0%)	–	8 (6.8%)	–	
Faculty–position					
Assistant professor	24 (35.3%)	24 (35.3%)	–	–	N/A
Associate professor	12 (17.6%)	12 (17.6%)	–	–	
Lecturer/senior lecturer	8 (11.8%)	8 (11.8%)	–	–	
Professor	24 (35.3%)	24 (35.3%)	–	–	
Faculty–years					
2–4 years	1 (1.5%)	1 (1.5%)	–	–	N/A
5–10 years	13 (19.1%)	13 (19.1%)	–	–	
>10 years	54 (79.4%)	54 (79.4%)	–	–-	
Specialty					
Allied health professional	7 (10.3%)	7 (10.3%)	–	–	N/A
Biochemistry	1 (1.5%)	1 (1.5%)	–	–	
Clinical genetics	1 (1.5%)	1 (1.5%)	–	–	
Dermatology	1 (1.5%)	1 (1.5%)	–	–	
Diagnostic specialties (radiology, pathology)	4 (5.9%)	4 (5.9%)	–	–	
Emergency medicine	4 (5.9%)	4 (5.9%)	–	–	
Hematology	1 (1.5%)	1 (1.5%)	–	–	
Medical education	1 (1.5%)	1 (1.5%)	–	–	
Medical specialties (cardiology, neurology, oncology, etc.)	19 (27.9%)	19 (27.9%)	–	–	
Non-medical doctor	3 (4.4%)	3 (4.4%)	–	–	
Ophthalmology	1 (1.5%)	1 (1.5%)	–	–	
Other (please specify):	1 (1.5%)	1 (1.5%)	–	–	
Otolaryngology	1 (1.5%)	1 (1.5%)	–	–	
Pediatric hematology oncology	1 (1.5%)	1 (1.5%)	–	–	
Pharmacology	2 (2.9%)	2 (2.9%)	–	–	
Physiologist	2 (2.9%)	2 (2.9%)	–	–	
Primary care (family medicine, internal medicine, pediatrics)	9 (13.2%)	9 (13.2%)	–	–	
Radiation oncology	1 (1.5%)	1 (1.5%)	–	–	
Research	1 (1.5%)	1 (1.5%)	–	–	
Surgical specialties	7 (10.3%)	7 (10.3%)	–	–	
Clinical work	49 (72.1%)	49 (72.1%)	–	–	N/A

**Figure 1:**
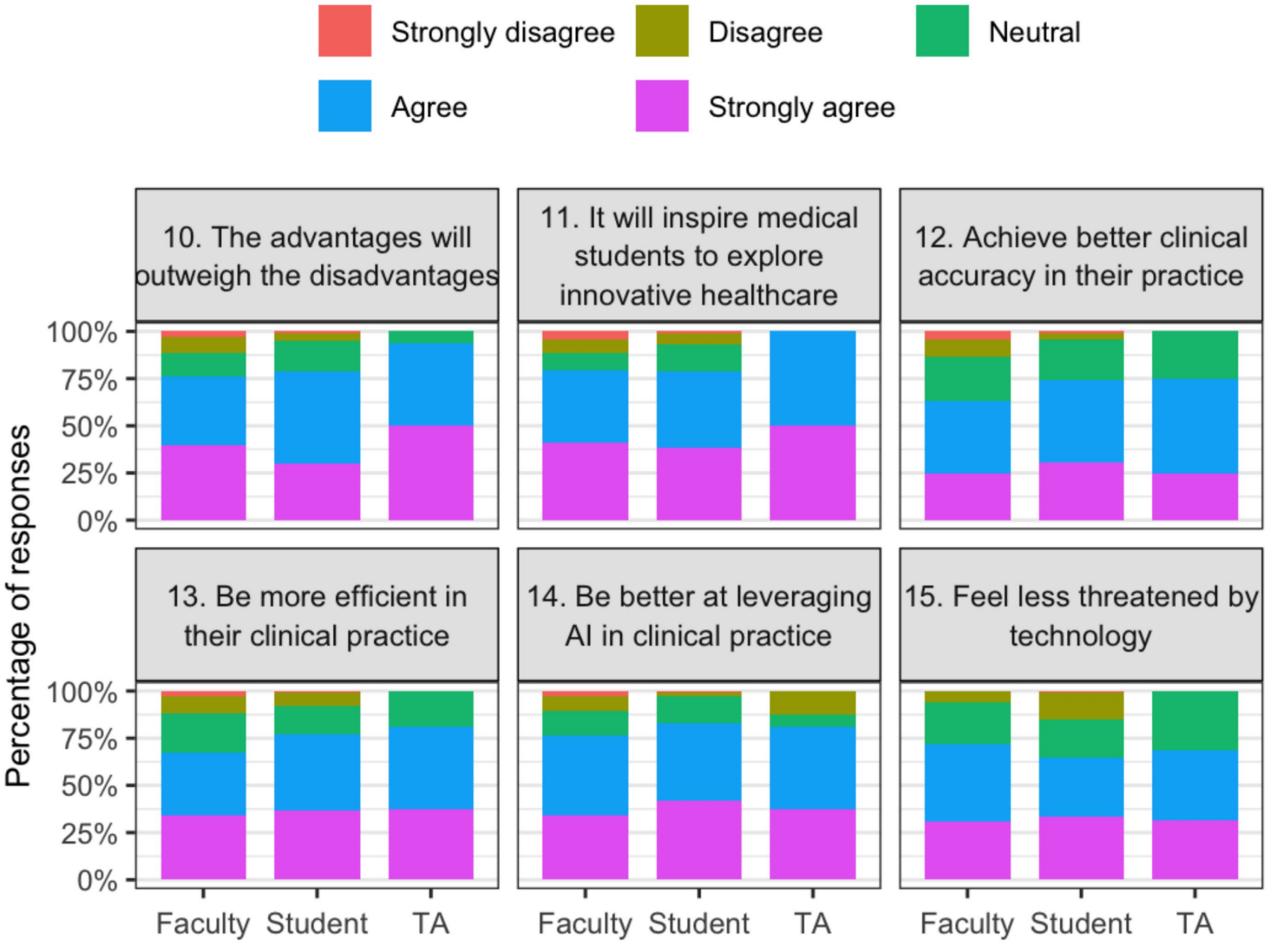
Perceived advantages of integrating artificial intelligence into the medical curriculum by role.

The scale’s internal consistency of the scale was found to be acceptable (Cronbach’s alpha of 0.76). Participants expressed generally favorable perceptions toward AI integration. Table 2 shows mean Likert scores by role. Respondents agreed that AI integration would inspire innovation in healthcare (mean 4.1 ± 1.0), enhance efficiency in clinical practice (mean 4.0 ± 1.0), and broaden medical specialties (mean 4.0 ± 0.9). They also agreed that AI applications will become common in healthcare (mean 4.2 ± 0.8). The most prominent concern was an increase in student workload (mean 3.3 ± 1.0). Faculty members, compared to students, expressed significantly greater concern about job replacement (mean 3.2 vs. 2.4, *p* < 0.001) and overreliance on technology (mean 2.4 vs. 1.9, *p* < 0.001). Figure 1 illustrates stacked Likert-scale response distributions for selected items reflecting perceived advantages of integrating artificial intelligence into the medical curriculum, stratified by participant role. Complete response distributions for all survey items, grouped by thematic domain and stratified by role, are provided in the Supplementary Figures S2, S3.

**Table 2:** AI questions as answered by study participants.

Variable	All *n* = 201	Faculty *n* = 68	Student *N* = 117	Teaching assistant *n* = 16	*p*-value
Questions mean ± sd					
The advantages will outweigh the disadvantages (Q10)	4.1 ± 0.9	4.0 ± 1.1	4.0 ± 0.8	4.4 ± 0.6	0.22
It will inspire medical students to explore innovative healthcare (Q11)	4.1 ± 1.0	4.0 ± 1.1	4.1 ± 0.9	4.5 ± 0.5	0.23
Achieve better clinical accuracy in their practice (Q12)	3.9 ± 0.9	3.7 ± 1.1	4.0 ± 0.9	4.0 ± 0.7	0.11
Be more efficient in their clinical practice (Q13)	4.0 ± 1.0	3.9 ± 1.1	4.1 ± 0.9	4.2 ± 0.8	0.34
Be better at leveraging AI in clinical practice (Q14)	4.1 ± 0.9	4.0 ± 1.0	4.1 ± 1.0	4.1 ± 0.9	0.21
Feel less threatened by technology (Q15)	3.9 ± 1.0	4.0 ± 0.9	3.8 ± 1.1	4.0 ± 0.8	0.55
Increasing medical student workload (Q16)	3.3 ± 1.0	3.4 ± 0.9	3.2 ± 1.1	3.4 ± 1.3	0.41
Potential resistance from students who are unfamiliar with AI technologies (Q17)	2.6 ± 1.0	2.9 ± 1.0	2.5 ± 1.0	2.6 ± 1.2	0.02
Resource limitations (Q18)	2.8 ± 1.1	3.0 ± 1.1	2.8 ± 1.1	2.4 ± 1.3	0.14
The fear that AI could replace medical jobs in the future (Q19)	2.6 ± 1.2	3.2 ± 1.2	2.4 ± 1.3	2.0 ± 1.0	<0.001
The possibility of an overreliance on technology in medical practice (Q20)	2. 0 ± 1.0	2.4 ± 1.1	1.9 ± 0.9	1.6 ± 0.6	<0.001
AI will overshadow the need for critical thinking and clinical reasoning skills (Q21)	2.1 ± 1.1	2.4 ± 1.2	2.0 ± 1.1	1.8 ± 0.8	0.01
Medical students will not need an advanced understanding of AI (Q22)	3.2 ± 1.2	3.4 ± 1.1	3.2 ± 1.2	2.8 ± 1.2	0.15
Medical education will fall behind if AI teaching modules are not integrated (Q23)	3.5 ± 1.1	3.5 ± 1.1	3.4 ± 1.1	4.1 ± 0.9	0.05
AI applications in medicine will become common (Q24)	4.2 ± 0.8	4.1 ± 0.9	4.2 ± 0.8	4.4 ± 0.5	0.38
AI will broaden some medical specialties (Q25)	4.0 ± 0.9	3.9 ± 1.0	4.0 ± 0.9	4.2 ± 0.7	0.53
AI will make medical care more accessible (Q26)	4.0 ± 1.0	3.8 ± 1.0	4.0 ± 1.0	4.2 ± 0.8	0.16
Overall score (0–100)	61.0 ± 11.5	62.7 ± 14.6	59.9 ± 9.7	61.2 ± 7.7	0.28
Stage in a medical student’s education that’s most suitable for integration of AI teaching module into medical curriculum					
Fellowship	9 (4.5%)	6 (8.8%)	3 (2.6%)	0 (0.0%)	0.54
Medical school clinical years	66 (32.8%)	20 (29.4%)	40 (34.2%)	6 (37.5%)	
Medical school preclinical years	112 (55.7%)	37 (54.4%)	66 (56.4%)	9 (56.3%)	
Residency	14 (7.0%)	5 (7.4%)	8 (6.8%)	1 (6.3%)	

When asked about the most appropriate stage for curriculum integration, more than half of the participants (55.7%) selected the preclinical years, while 32.8% preferred the clinical years. Only 7.0 and 4.5% indicated residency and fellowship, respectively, as the best stages for integration.

Multiple linear regression analyses for AI scores are presented in Table 3. Variables included in the model were age, gender, nationality and position. None of these factors were found to be statistically significantly associated with overall perception scores. However, participants aged 45 years or older had higher scores compared to those aged 18–24 (*β* = 6.82, 95% CI –2.11 to 15.75, *p* = 0.13). Moreover, males, non-Saudis and students had also non-significant higher scores.

**Table 3:** Multiple linear regression for the AI scores (based on all 17 questions).

Variable	Grouping	Beta	Confidence interval (95%)	*p*-value
Age	18–24	REF	REF	REF
	25–44	2.96	(−3.7, 9.70)	0.39
	45+	6.82	(−2.11, 15.75)	0.13
Gender	Male	REF	REF	REF
	Female	−2.21	(−5.68, 1.26)	0.21
Nationality	Saudi	REF	REF	REF
	Non-Saudi	0.47	(−3.56, 4.50)	0.82
Position	Faculty	REF	REF	REF
	Student	3.53	(−4.8, 11.95)	0.41
	Teaching assistant	2.67	(−6.23, 11.57)	0.56

Multiple logistic regression analyses for positive perception of AI integration are presented in Table 4. Same variables are included in this model, mainly age, gender, nationality and position. Also, none of these factors were found to be statistically significantly associated with positive perception of AI integration. Those who were aged 45 year and older were more likely to have positive perception of AI integration (OR = 1.56, 95% CI 0.31–7.88, *p* = 0.59). Similarly, males and students were more likely to have positive perception of AI integration.

**Table 4:** Multiple logistic regression predicting positive perception of AI integration (overall AI score ≥60 vs. < 60).

Variable	Grouping	Odds ratio	Confidence interval (95%)	*p*-value
Age	18–24	REF	REF	REF
	25–44	1.56	(0.45, 5.43)	0.48
	45+	1.56	(0.31, 7.88)	0.59
Gender	Male	REF	REF	REF
	Female	0.58	(0.32, 1.08)	0.09
Nationality	Saudi	REF	REF	REF
	Non-Saudi	1.00	(0.49, 2.07)	0.98
Position	Faculty	REF	REF	REF
	Student	1.19	(0.26, 5.48)	0.83
	Teaching assistant	0.93	(0.19, 4.58)	0.93

Outcome variable: overall AI perception score (0–100), dichotomized at ≥60 to indicate positive perception.

## Discussion

This study evaluated perceptions of integrating AI teaching modules into the undergraduate medical education curriculum at Alfaisal University, recognizing its potential in enhancing patient medical care. Participants included Alfaisal faculty, students, and teaching assistants to reflect generational and role-based differences in perception. Overall, participants expressed positive perceptions toward integrating AI into the medical curriculum, with high agreement that AI can inspire innovation, improve efficiency, broaden medical specialties, and become common in healthcare. These findings are consistent with prior studies across different regions, where both students and faculty acknowledged AI’s potential to enhance medical education and clinical practice (9).

Most participants preferred its integration into preclinical years as compared to clinical years, with very few preferring its integration into residency and fellowship. This reflects the belief that AI literacy should be established early on, allowing students to be better equipped to handle technological tools in patient care. Studies have shown promising outcomes of AI in medical education (20), emphasizing the importance of introducing AI fundamentals during preclinical years to build students’ familiarity and prepare them for the effective use of AI tools in clinical settings.

While there were many positive perspectives, certain concerns remained significant. Specifically, concerns about job replacement and overreliance on technology were noted, and these concerns were notably higher among faculty members compared to students. This difference may be explained by the faculty’s greater clinical experience and awareness of system changes, whereas students are more native to the digital world and are more comfortable with technological integration. This has also been noted in other studies in the region, where physicians expressed concerns about job security and the potential for an emotional disconnect in patient care (7). All in all, faculty development programs that include AI workshops would bring us one step closer to addressing these concerns and start working on overcoming them.

Future studies should focus on comparisons between different universities, longitudinal studies to assess the impact of AI training on clinical reasoning, and be more specific in their population to obtain more targeted evidence. They can also conduct comparative studies between populations from different regions within the kingdom; this will not only provide data related to regional awareness but also shed light on important areas for development. This study has several limitations. Since the study was conducted in a non-profit university, generalizability can be limited. The cross-sectional nature limits any causal inferences. However, the study’s novelty lies in its limited focus on AI integration in medical education.

## Conclusion

The study highlights the potential benefits of introducing artificial intelligence early in Alfaisal University’s undergraduate medical program. While students show strong enthusiasm for incorporating AI into their education, faculty members emphasize the importance of addressing potential challenges, such as increased workload, concerns about job displacement, and over-reliance on technology. These findings underscore the need for a thoughtful and balanced integration of AI modules, ensuring that future physicians are equipped with the skills and competencies required to thrive in an evolving, technology-driven healthcare landscape.

## Data Availability

The raw data supporting the conclusions of this article will be made available by the authors, without undue reservation.
