# Supplementary material for: Perception of integrating an AI teaching module into medical education curriculum
Source: Front Med (Lausanne). 2026 Mar 3;13:1774935. doi: 10.3389/fmed.2026.1774935 (PMC12992059; doi:10.3389/fmed.2026.1774935)
Supplement: Supplementary file 1 [file Data_Sheet_1.pdf]

Supplementary Figure S2. Perceived disadvantages and challenges of integrating artificial intelligence into the medical curriculum by role

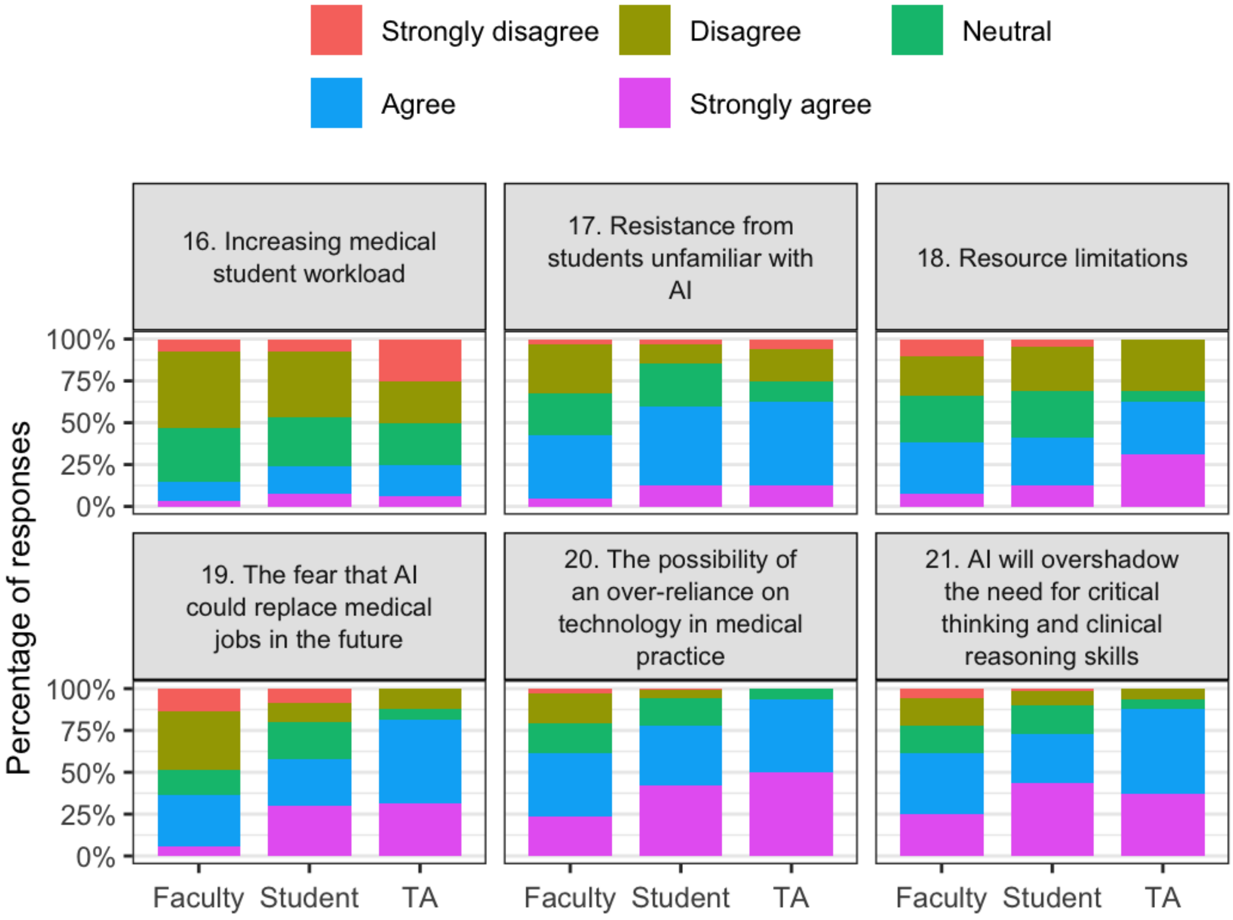

Stacked Likert-scale distributions illustrating participants' agreement with statements describing perceived disadvantages and challenges of integrating artificial intelligence (AI) into the medical curriculum (Q16–Q21), stratified by role (faculty, students, and teaching assistants).

TA = teaching assistant.

Supplementary Figure S3. Perceptions of the future role of artificial intelligence in medicine and medical education by role

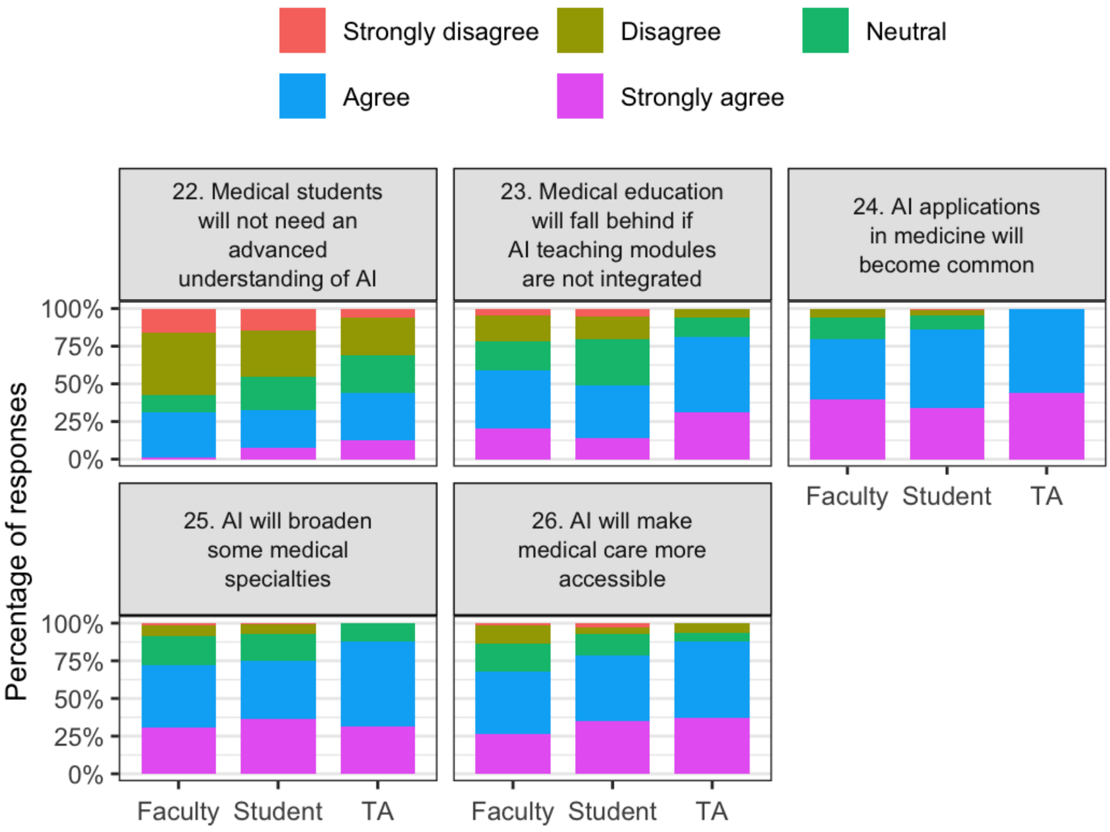

Stacked Likert-scale distributions showing participants' perceptions regarding the future role of artificial intelligence (AI) in medicine and medical education (Q22–Q26), stratified by role (faculty, students, and teaching assistants).
TA = teaching assistant.
